# The Contribution of Physical Exercise to Brain Resilience

**DOI:** 10.3389/fnbeh.2020.626769

**Published:** 2021-01-20

**Authors:** Ricardo Mario Arida, Lavinia Teixeira-Machado

**Affiliations:** ^1^Department of Physiology, Federal University of São Paulo, São Paulo, Brazil; ^2^Department of Education in Health, Federal University of Sergipe, Sergipe, Brazil

**Keywords:** brain reserve, cognitive reserve, physical activity, exercise, neurological disorders, neuroprotection, stress, brain resilience

## Abstract

Increasing attention has been given to understanding resilience to brain diseases, often described as brain or cognitive reserve. Among the protective factors for the development of resilience, physical activity/exercise has been considered to play an important role. Exercise is known to induce many positive effects on the brain. As such, exercise represents an important tool to influence neurodevelopment and shape the adult brain to react to life's challenges. Among many beneficial effects, exercise intervention has been associated with cognitive improvement and stress resilience in humans and animal models. Thus, a growing number of studies have demonstrated that exercise not only recovers or minimizes cognitive deficits by inducing better neuroplasticity and cognitive reserve but also counteracts brain pathology. This is evidenced before disease onset or after it has been established. In this review, we aimed to present encouraging data from current clinical and pre-clinical neuroscience research and discuss the possible biological mechanisms underlying the beneficial effects of physical exercise on resilience. We consider the implication of physical exercise for resilience from brain development to aging and for some neurological diseases. Overall, the literature indicates that brain/cognitive reserve built up by regular exercise in several stages of life, prepares the brain to be more resilient to cognitive impairment and consequently to brain pathology.

## Introduction

Stressful life events can have a considerable impact on brain function and structure, resulting in the development of several psychiatric disorders (Ludwig et al., 2018; Chow and Choi, 2019). Interestingly, most individuals do not develop such illnesses after experiencing stressful life events and are thus thought to be resilient. Resilience is defined as the capacity to adapt successfully to acute stress, trauma, or chronic adversity (Russo et al., 2012). In this context, researchers have introduced the concept of brain resilience, often described as brain reserve or cognitive reserve (Medaglia et al., 2017). Brain reserve is related to the structural properties of the brain (brain volume, the number and size of neurons, cortical thickness) and cognitive reserve refers to a process where the brain copes with brain pathology. Reserve of either type expresses alteration in the function or structure of the brain that modifies cognitive and behavioral capacities following brain damage (for review, see Medaglia et al., 2017). Some key brain structures involved in specific generation and regulation of emotional, cognitive, and behavioral responses to stressors include insula, nucleus accumbens, amygdala, hypothalamus, hippocampus, medial prefrontal, and anterior cingulate cortex (Gupta et al., 2017) and dysregulation in these circuits has been related to emotional distress, anxiety, and mood disorders (Gupta et al., 2017; Iadipaolo et al., 2018).

Changes in these neural circuits affect numerous neurotransmitter systems and molecular pathways. Among them, hormones, neuropeptides, neurotransmitters systems, and neurotrophic factors are involved in the responses to stress. Alterations in their functions determine the individual variability in stress resilience (Feder et al., 2009; Russo et al., 2012). For instance, early life stress has been linked to long-lasting changes in the hypothalamus-pituitary-adrenal axis, resulting in chronically high levels of corticotrophin releasing hormone and cortisol. These events usually lead to structural changes in brain regions such hippocampus and amygdala (McEwen and Milner, 2007). Stress also induces the release of noradrenaline and serotonin in several brain areas associated to anxiety disorders and mood regulation, and dopamine in response to aversive or rewarding stimuli. Thus, considering the involvement of neurotrophins in the regulation of different forms of normal and pathological behavior, they play a critical role in resilience to chronic stress (Taliaz et al., 2011; Rothman and Mattson, 2013). Positive adaptive changes in these systems are suggested to promote resilience (Feder et al., 2009). In this regard, adaptive coping strategies in stressful situations, such as neurofeedback training have been used to reduce depressive symptoms and improve emotion regulation (Keynan et al., 2019) and a relationship between functional network at rest and resilience has been evidenced (Paban et al., 2019).

Studies have used resilience measurement scales to be applied in general and clinical populations and the Connor–Davidson Resilience Scale (CD-RISC) presents suitable psychometric properties and allows for valuable measurement of resilience (Campbell-Sills and Stein, 2007; Smith et al., 2008; Windle et al., 2011). Adapted version of brief resilience scale has been large used to this purpose.

Brain resilience or vulnerability following a stressor is influenced by innate difference (genetically determined) and the experiences or exposures of an individual across the life span (education, occupation, engagement in physical/sport activities, or social activities) (Stern et al., 2018). Therefore, the interplay between genetic predisposition and lifestyle factors has a critical role in determining resilience to brain disorders (Walhovd et al., 2019). Genetic variation, resulting alterations in neurotransmitter systems have been of particular interest for brain resilience. Differences between individuals possible reflect the interaction between genetic predisposition, early-life experiences and experiences across the lifespan. Accordingly, environmental stimuli during the early life period can exert prominent effects on the risk of neurological disorders (Lesuis et al., 2018). In this regard, a considerable number of scientific publications have reported that the combination of both physical and cognitive stimulation can affect brain resilience (Deuster and Silverman, 2013; Bozzali et al., 2015; Pedrinolla et al., 2017; Casaletto et al., 2020). The literature has clearly demonstrated that an active lifestyle is inversely associated with stress-related health problems resulting in the development of chronic diseases (Deuster and Silverman, 2013; Clark et al., 2019). Thus, healthy behaviors obtained early in life and maintained throughout a long period of life can build brain resilience against age-related diseases (Gale et al., 2012; McEwen, 2016; Lesuis et al., 2018).

**Figure 1:**
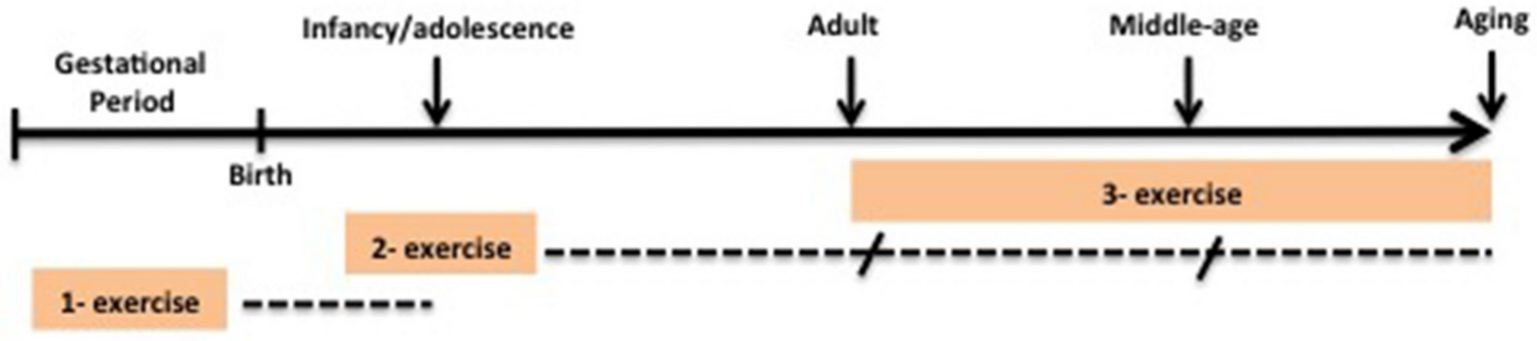
Design of intervention studies on brain resilience in this review. Dotted lines represent follow up outcomes. (1). Exercise during pre-natal period and its effect on brain offspring (infancy and adolescence). (2) Exercise during post-natal brain development (infancy and adolescence) and its effect in later life. (3) Exercise in adulthood, aging and in some-neurologic disease.

Several key positive factors that occur from childhood or adolescence through to adulthood contribute to brain resilience (Burt and Paysnick, 2012). Among them, physical activity/exercise has an important role. Indeed, in many publications on resilience, one frequently mentioned factor for promoting resilience is physical exercise/fitness (Wu et al., 2013; McEwen, 2016). The beneficial influence of physical activity/exercise on resilience can in part be attributed to the understanding that it can induce positive physiological and psychological improvements, protect against the effects of stressful events, and prevent or minimize several neurological diseases. In this sense, this review will consider the following question with the target population being all life stages, including some neurological disorders, using physical exercise as intervention and brain resilience as outcome: How can physical exercise contribute to brain resilience in all life stages and in some neurological disorders? Here, we outline data generated from clinical and pre-clinical studies and discuss the possible biological mechanisms underlying the positive impact of physical exercise on brain resilience in the following conditions: (1) exercise during the pre-natal period and its effect on offspring's brain (infancy and adolescence), (2) exercise during post-natal brain development (infancy and adolescence), and (3) exercise in brain adulthood, aging, and in some neurological diseases (Figure 1). We focused on the impact of exercise on psychosocial stressors with regard to educational (cognitive performance), professional, and physiological performance.

### Search Strategy

The literature search was conducted between February and August 2020. A comprehensive PubMed search was conducted to identify studies investigating the role of exercise in brain resilience. The following terminology was applied: (“exercise” OR “physical activity” OR “sport”) AND (“stress” OR “resilience” OR “brain resilience” OR “cognitive reserve” OR “brain reserve” OR “neural reserve”) AND (“neurobiological factors” OR “endocrine system” OR “genes” OR “genetic” OR “epigenetic”) AND (“psychiatric disorders” OR “depression” OR “anxiety” OR “brain diseases”). Written in English, original studies, letters as well as reviews were included. Studies identified were selected and excluded by examining the titles, abstract, or full text articles. Studies that did not address physical activity/exercise for brain resilience in either clinical or pre-clinical studies were excluded.

The terms physical activity, exercise, and physical fitness are often used in the literature, and for clarification it is important to differentiate these denominations. For instance, physical activity has been used interchangeably with the term exercise.” The frequently cited article by Caspersen et al. (1985) defines physical activity as any bodily movement produced by skeletal muscles which results in energy expenditure, while exercise is a physical activity that is planned, structured, repetitive, and purposeful. In contrast with physical activity, physical fitness is characterized by a number of components that an individual achieves related to health, particularly cardiorespiratory endurance, body composition, muscular strength, and flexibility. Being physically active is not necessarily the same as having high aerobic fitness.

## Results

Of the 147 potentially relevant articles from the PubMed database, 12 studies addressed physical exercise for brain resilience. Most were excluded because they did not address physical activity/exercise (*n* = 92) or the brain resilience outcome (*n* = 43). To conduct a scoping review looking at all life stages and some neurological conditions, both clinical and pre-clinical research, we included 114 more studies through a free search on pregnancy (*n* = 9), childhood and adolescence (*n* = 18), adulthood and aging (*n* = 16), Alzheimer's disease (*n* = 17), Parkinson's disease (*n* = 14), Epilepsy (*n* = 20), Stroke (*n* = 16), systematic reviews (*n* = 3), and clinical guidelines (*n* = 1).

## Discussion

For didactic reasons, we will present the translational contribution of physical exercise to brain resilience and in neurobiological conditions in the following order: physical exercise in pregnancy, in childhood and adolescence, in adulthood, aging, and neurological disorders.

### Physical Exercise in Pregnancy

Brain development consists of a complex interaction of molecular, cellular, and environmental systems. It begins in the third gestational week and continues at least through late adolescence. The developing brain is constantly influenced by internal and external factors and certain events occurring during this period may positively or negatively affect the brain's development (Andersen, 2003). With respect to this scenario, evidence has shown that environmental stimuli such as physical activity/exercise can impact positively on brain development (Clapp, 1996; Esteban-Cornejo et al., 2015). Investigations on the influence of exercise at earlier stages of brain development have been encouraging, including exercise interventions on pre- and post-natal brain development.

In humans, a limited number of studies has examined whether exercise during the maternal period can influence offspring. A classic study conducted over 25 years ago demonstrated a better performance on tests of general intelligence and oral language skills in children aged 5 years from mothers who exercised during pregnancy (Clapp, 1996). In another investigation, physical activity before and during gestation positively influenced offspring's academic performance in youth (from 6 to 18 years) (Esteban-Cornejo et al., 2015). These outcomes suggest that routine exercise before and during the pre-natal period may produce benefits for infants' and adolescents' academic performance. However, whether pre-natal exercise contributes to brain resilience at later stages of life has not been explored in humans. Animal studies have provided promising data and are highlighted elsewhere in this review.

In contrast with restricted investigations in humans, a substantial number of animal studies has examined the influence of exercise during the maternal period on offspring. Evidence from laboratory animals has shown that regular physical exercise during pregnancy can affect several aspects of cognition and behavior in offspring. These findings result from experiments using different models of physical exercise, including forced treadmill running, forced swimming, and voluntary wheel running. For instance, the swimming or treadmill running exercise during pregnancy improves spatial learning (Parnpiansil et al., 2003) and short-term memory in offspring (Lee et al., 2006; Kim et al., 2007) and voluntary activity reduces depression-like behavior and increased learning and memory performance in offspring (Bick-Sander et al., 2006). The influence of exercise during the gestational period is not restricted to the offspring's cognitive functions. For instance, an acceleration of visual system plasticity was detected through earlier eye opening and visual acuity in pups from dams who lived in an enriched environment seven days before delivery (Cancedda et al., 2004). These findings indicate that pre-natal sensory-motor stimulation induces accelerated brain maturation in early post-natal life.

Another question to be clarified is whether the effects of exercise during pregnancy can be sustained into adulthood. In this regard, Robinson and Bucci (2014) examined the effect of voluntary wheel running during the gestational period in adult offspring. Improvement in object recognition memory was observed in progenies from 60 to 88 days of age. In another study, treadmill running during pregnancy promoted enhanced cognitive function (habituation behavior and spatial learning) in adult offspring (Gomes da Silva et al., 2016), indicating that exercise during the peri-natal period may increase brain function in offspring in adult life. Therefore, positive early life experiences such as physical exercise during the gestational period may persist in offspring and may consequently play an important role in increasing brain resilience in later life.

### Neurobiological Mechanisms

Considering that few studies in humans have examined the effects of physical activity during pregnancy on offspring's neurodevelopment, the mechanism by which exercise during pregnancy impacts on progeny's cognitive function is just starting to be clarified. Two studies conducted by Clapp (1996) and Clapp et al. (1998) focusing on the first years of life (ages one and five), found no significant difference in the offspring's morphometric parameters (head circumference and height). In a more recent investigation, a better cerebral maturation in the newborn of exercised pregnant mothers was observed by means of a neurophysiological index of sound discrimination and auditory memory (the slow positive mismatch response—SPMMR) (Labonte-Lemoyne et al., 2017). The long-term effects of exercise during pregnancy on the offspring, i.e., in their adult life, still remain to be elucidated.

Unlike human studies, much more information has been collected from laboratory animals and several factors have been proposed on these effects. Prominent cell proliferation and survival in offspring following exercise during pregnancy have been observed (Bick-Sander et al., 2006). For instance, swimming (Lee et al., 2006) and treadmill running (Kim et al., 2007) from embryonic day 15–21, the late developmental stage of the rodent fetus, provoked an increase in the number of BrdU-labeled cells (marker of proliferating cells) on post-natal day 29 (P29) in the hippocampus, a region related to learning and memory, and this proliferative effect was associated with an improvement of cognitive function. Significant changes may also occur in later periods of life. Some studies have shown an increase in absolute numbers of neuronal and non-neuronal cells and brain-derived neurotrophic factor (BDNF) levels in the hippocampal formation of adult offspring (P60) (Gomes da Silva et al., 2016) and increased cell density in the hippocampal CA1 and CA3 areas during the adult period (P120) (Dayi et al., 2012) of offspring of mothers trained during the gestational period. Of note, increased BDNF levels in the hippocampal formation of pups of exercised mothers were associated with better spatial learning (Akhavan et al., 2008) and BDNF deletion resulted in learning deficits (Gorski et al., 2003). With regard to this, molecular systems, essentially involving BDNF, have been reported to play an important role in exercise-induced cognitive improvement. Overall, offspring of exercised mothers during pregnancy show increased neurogenesis, hippocampal BDNF mRNA and BDNF protein, and improved learning and memory throughout life (Lee et al., 2006; Kim et al., 2007; Gomes da Silva et al., 2016), indicating that increased BDNF expression induced by exercise during gestation may improve brain function in the offspring.

The results indicate that pre-natal exercise can induce long-term effects on the hippocampal plasticity of progeny (Dayi et al., 2012). These findings are of great importance because the hippocampal region contributes to long-term potentiation (LTP), one of the cellular mechanisms required for learning and memory (Bliss and Collingridge, 1993). It is well-known that activation of the N-methyl-D-aspartate (NMDA) glutamate receptor generates LTP, as well as learning and memory. In this context, better performance in the Morris water maze tests was observed in pups of trained mothers during pregnancy compared with pups of sedentary mothers and this effect was abolished by the NMDA antagonist MK-801 (Akhavan et al., 2008). In addition to the cognitive effects, there is supportive evidence that exercise during pregnancy exerts a neuroprotective effect in the progeny. In the study by Herring et al. (2012), voluntary wheel running during pregnancy reduced beta-amyloid plaque burden, APP processing, oxidative stress, inflammation, and neurovascular dysfunction in offspring at 5 months of age, suggesting that pre-natal physical activity may also contribute to reducing the future risk of neurological diseases.

### Physical Exercise in Childhood and Adolescence

In relation to exercise interference in post-natal brain development, i.e., during infancy or adolescence, numerous human studies have reported positive findings in cognitive functioning following acute (Hillman et al., 2009; Ellemberg and St-Louis-Deschênes, 2010) or chronic physical exercise (Hillman et al., 2005, 2014). A meta-analysis of 16 studies showed a positive association between cognition (learning and intelligence scores) and physical activity in school-age children (Sibley and Etnier, 2003). A superior performance in three stroop conditions (attention tasks) was observed in children between seven and 12 years of age with better aerobic fitness (Buck et al., 2008) and in arithmetic cognition in fitter pre-adolescent children aged nine–ten (Moore et al., 2014). An elegant investigation conducted by a Swedish group who evaluated 1,200,000 adolescents showed a positive association between physical fitness and better cognitive performance at age 18 years and occupational status and educational achievement later in life (Aberg et al., 2009). A further important consideration is whether regular physical activity during adolescence or adult life promotes long-lasting cognitive effects. In the study by Dik et al. (2003), physical activity at 15 and 25 years induced better cognitive performance at 62 and 85 years of age. These findings indicate that regular physical activity in early life is able to improve cognitive functioning, supporting the cognitive reserve hypothesis. Although very optimistic, confirmatory studies have yet to be conducted in further human studies.

While promising findings on the effects of exercise during infancy or adolescence have been well-documented, far less is known about its impact on brain resilience. Chronic stress during the adolescent period may impact negatively, both physically and psychologically (Sheth et al., 2017) and regular physical activity may be of great value in adolescent stress management. For instance, Norris et al. (1992) conducted a retrospective and experimental study to examine the influence of physical activity and an exercise program on psychological stress and well-being in adolescents. In their retrospective analysis, adolescents who engaged in exercise/sports activities more regularly reported less perceived stress and depression. In the second part of their study, a group of adolescents submitted to a regular exercise program reported significantly less perceived stress than their controls, suggesting that physically fit adolescents seem to be less susceptible to life stressors. In a recent cross-sectional survey (Kim et al., 2019), adolescents from 14 to 19 years old who engaged in physical activities more than five times per week were stressed less often than those who were involved in fewer physical activities. To indicate whether any psychological benefits would have been maintained, it would be reasonable to analyze a follow-up period at the end of the physical exercise program or physical activity. Of note, a cross-sectional study that examined the relationship between participating in physical activity at several time-points in life (teenage, age 30, age 50, and late-life) and late-life cognitive function showed that teenage physical activity was greatly associated with a lower probability of cognitive impairment in elderly people who were physically active (Middleton et al., 2010). However, the survey did not take account of whether cognitively impaired subjects had been through stressful life events. Thus, conditions other than a stressful early life may influence cognitive reserve in later life, such as nutritional status during childhood (Zhang et al., 2010) and education (Zahodne et al., 2015). Overall, these findings suggest that the cognitive reserve built up in physically fit subjects in the early stages of life prepare the brain to be more resilient to cognitive impairment, dementia, and consequently Alzheimer's pathology; this probably occurs through brain plasticity.

In addition to cross-sectional studies, we should note how long-term physical activity can impact in later life. A prospective 6-years follow-up study with 496 adolescent girls designed to examine the relationship between physical activity and mental health during adolescence showed a reduced risk for future depressive symptoms (Jerstad et al., 2010). In another prospective and retrospective analysis, physical activity during childhood and adolescence was associated with a reduced risk of depression 20 years later (McKercher et al., 2014). Subsequently, a long-term follow-up over a period of 14 years, using a large sample of subjects from adolescence through adult life (18–29 years old), showed that those engaged in higher levels of physical activity presented lower levels of depressive symptoms over time (from adolescence into adulthood) (McPhie and Rawana, 2015). Collectively, the scientific literature presents strong indicators that habitual physical/sport activities at an early age may boost lifelong brain function and may also reduce the risk of several brain disorders.

Similar findings have been found in laboratory animals. In a classic study conducted by Uysal et al. (2005), young rats submitted to treadmill exercise during adolescence and early adulthood (from post-natal day 22 to post-natal day 78; P22 to P78) presented better spatial memory in the water maze test compared to non-exercised rats during this period. Using another exercise model (voluntary wheel running) and a non-spatial form of learning and memory (object recognition memory), it was observed that rats with free access to wheel running from P25 to P55 had a better discrimination ratio between novel and familiar objects compared to their controls (Hopkins et al., 2011). Notably, 2 and 4 weeks after the exercise had terminated, exercised rats performed better in the re-test compared to their controls. Similar findings were observed in a study conducted by our research group (Gomes da Silva et al., 2012). Treadmill running during the adolescent period (P21–P60) resulted in improved spatial learning and memory tested at P60–P65, and, like the study by Hopkins et al. (2011), rats showed better spatial memories in later life (at P96) (Gomes da Silva et al., 2012). These findings also indicate that physical exercise during this critical period of brain development can result in sustained cognitive benefits, that is, in a neural reserve that can be useful in old age.

### Neurobiological Mechanisms

Changes in function and brain structure are clearly associated with exercise-induced improvement in cognition early in life. Improved fronto-temporal white matter integrity, which is related to improved cognitive function, has been observed following 8 months of aerobic exercise in overweight 8–11 year-old children (Schaeffer et al., 2014). Greater hippocampal volume and better performance in relational memory were detected in fitter children compared to less fit children (Chaddock et al., 2010). Also, greater gray matter volumes in frontal, temporal, and subcortical areas were also related to higher cardiorespiratory fitness; and some regions such pre-motor cortex, supplementary motor cortex, and hippocampus were related to better academic performance (Esteban-Cornejo et al., 2017). On the electro-physiological level, positive changes in event-related brain potential that may underlie cognitive performance have been observed in pre-adolescents following an acute bout of treadmill walking (Hillman et al., 2009) or in fitter children (Moore et al., 2014). Other factors by which physical exercise can interfere beneficially for cognition include the release of growth factors such as BDNF, Insulin-like growth factor 1 (IGF-I), and vascular endothelial growth factor (VEGF), possibly providing reserve against later cognitive decline and dementia. These neurotrophins are produced and secreted in the brain and play an essential role in regulating proliferation, development, and cellular differentiation. For instance, an aerobic exercise program increased serum BDNF levels in adolescents, which was associated with better working memory (Jeon and Ha, 2017). A single bout of high-intensity, but not low-intensity, exercise was able to improve memory as shown by remembering more newly learned vocabulary words compared to the control group (Hötting et al., 2016). Supporting the above findings, a recent systematic review and meta-analysis reported significant results on the levels of BDNF and executive function in adolescents submitted to physical exercise (de Azevedo et al., 2019). Overall, the combined effects of neurotropic factors and physical exercise from childhood to adolescence, a critical window of brain development, may contribute to improvements in memory and also in various aspects of academic performance, consequently preventing or minimizing age-associated cognitive decline.

With regard to the results of animal studies during post-natal brain development, increased hippocampal cell proliferation was shown in rats trained over five consecutive days with 4-week-old (Kim et al., 2004) and neurogenesis in rats trained for one week from P29 to P35 (Lou et al., 2008). Our research group observed that increased hippocampal cell proliferation (stained with Ki67) induced by exercise occurred mainly in the earliest stages of post-natal development (de Almeida et al., 2013). In line with this, Uysal et al. (2005) reported an association between an increased number of hippocampal cells in CA1, CA3, and the dentate gyrus and better spatial memory in trained rats during development. Improvement in spatial memory was also related to increased axonal density of granule cells in the dentate gyrus stained by the Neo-Timm method in trained adolescent rats (Gomes da Silva et al., 2012). As highlighted previously, BDNF plays a potential role in the modulation of cognitive functions. To this end, higher BDNF mRNA (Abel and Rissman, 2013), BDNF expression, and its receptor tropomyosin-related kinase B (Gomes da Silva et al., 2012) have been detected in rats trained during adolescence, indicating that brain BDNF in early life induced by exercise may be associated with cognitive improvement.

### Physical Exercise in Adulthood, Aging, and Neurological Disorders

As outlined, positive early life experiences or lifestyle factors such as physical exercise have been associated with resilience in later life, suggesting that regular exercise can generate brain protection by means of reserve. For adult, middle-aged or aged people, physical exercise is frequently recognized to slow cognitive decline and protect against the consequences of stressful events. For instance, the practice of physical exercise in mid-life (from age 25–50) was able to reduce the chance of dementia in older adults (Andel et al., 2008) and improve cognition, mainly for executive-control processes (Colcombe and Kramer, 2003).

Despite the growing number of positive findings relating exercise and cognition in adulthood and aging, some prospective longitudinal studies have reported null results (Broe et al., 1998; Wilson et al., 2002). In the study by Broe et al. (1998), no association was found between exercise and cognitive performance, and in another survey, cognitive but not physical activities were associated with a reduced risk of cognitive decline (Wilson et al., 2002). We have to bear in mind that mixed findings related to the effect of physical activity in cognition can be partly attributed to cognitive reserve. Sánchez Rodríguez et al. (2011) used factor scores to assess cognitive reserve, classifying the participants into high or low reserve. Patients with Alzheimer's disease (AD) with low cognitive reserve scores presented more deficits in neuropsychological tests such as memory, attention, and language than patients with high cognitive reserve. Higher education, higher professional performance, and ludic activities were factors contributing to cognitive reserve and physical activity was not considered a factor for cognitive reserve in their study. Similarly, Fritsch et al. (2007) found educational level but not physical and social activities to be predictors of cognitive function in the elderly.

The above studies suggested that although the cognitive reserve is influenced by intrinsic differences or during childhood, stimuli at later periods of life can also positively or negatively alter the reserve. Thus, some variables such lifestyle habits, age, different follow-ups, physical exercise habits, or types of exercise intervention are confounders that can interfere with the studies' outcomes. To deal with any research gaps, a growing number of meta-analyses have been conducted to verify the impact of physical activity in the adult or elderly population. For instance, individuals aged ≥40 years with better levels of physical activity presented lower risk of cognitive decline and dementia (Blondell et al., 2014). An investigation by Hamer and Chida (2009) using a meta-analysis of prospective studies found that habitual physical activity was able to reduce by 28% and 45% the risk of dementia and the risk of AD, respectively. In a more recent meta-analysis, exercise duration of 45–60 min per session and of moderate or vigorous intensity was associated with improvement in cognition function in subjects older than 50 years (Northey et al., 2018). Since age is the main risk factor for dementia, particularly Alzheimer's, sustained physical/sports activities have been associated with reduced incidence of AD and therefore increased resilience against developing AD (Rovio et al., 2005; Lautenschlager et al., 2008).

Studies have demonstrated that psychological stress and other psychosocial factors such depression increase vulnerability to AD (Mejía et al., 2003; Aznar and Knudsen, 2011; Yuede et al., 2018) and regular physical exercise plays an important role in stress management and increased resistance to stress-related disorders (Tsatsoulis and Fountoulakis, 2006). Although studies have examined the effects of stress or physical exercise on AD independently, the interaction of stress and exercise needs to be better explored. To this end, a review by Yuede et al. (2018) reported attractive findings from pre-clinical studies on this interface.

AD has also been associated with the occurrence of cerebrovascular disease and physical exercise plays a positive role in this condition (Lange-Asschenfeldt and Kojda, 2008). Physical exercise of moderate intensity increases cerebral blood in humans (Braz and Fisher, 2016), which could minimize the effects of hypoperfusion in AD. Hypotheses by Nation et al. (2011) have been formulated concerning the impact of exercise and stress on the cerebrovascular system with increased risk of developing AD, highlighting preventive strategies for this disease.

In addition to the preventive effect described above, physical activity/exercise has been demonstrated to promote neurobiological benefits for brain pathology, consequently attenuating the decline in AD. Authors such Hoffmann et al. (2015) demonstrated that a supervised exercise program over 16 weeks was able to delay neuropsychiatric symptoms in patients with mild AD. In a subsequent investigation from the same research group, using the same exercise protocol, a positive correlation was found between frontal cortical volume and measures of mental speed and attention (Frederiksen et al., 2018). Cardiorespiratory fitness, as measured by maximal oxygen consumption (VO2max), has been associated with cognition (Wendell et al., 2014) and this evidence has also been reported in patients with AD (Vidoni et al., 2012). In line with this, a study analyzing a direct measure of VO2max in patients with mild AD detected a positive connection between VO2 peak, cognition, and neuropsychiatric symptoms (Sobol et al., 2018). In sum, an increasing number of studies have reinforced the role of exercise in minimizing the reduced cognitive function in AD (Groot et al., 2016).

In animals, extensive literature has shown a positive association between exercise and cognition in adulthood and aging, supporting the important role of exercise in brain health as demonstrated here. Classic studies by van Praag et al. reported that voluntary wheel running improved hippocampus-dependent learning and memory in young adult (van Praag et al., 1999) and aged mice (van Praag et al., 2005). Similar findings have also been demonstrated in other exercise protocols. Results from forced and voluntary exercise showed improved short-term and spatial memories in young and old rats (Kim et al., 2010; Speisman et al., 2013) and resistance exercise improved performance in the passive avoidance test, a hippocampus-dependent memory task, in young adult rats (Cassilhas et al., 2012). With regard to whether exercise can impact positively in stressful conditions, a recent study showed that impaired cognition induced by chronic stress in mice was prevented in stressed animals submitted to regular moderate and intense exercise (Lee et al., 2018). This adaptive effect of regular exercise on the homeostatic system can improve resistance and/or resilience to physical and psychological stress and may consequently protect against brain disease. To this end, exercise can play an important role in increasing resistance to stress in AD. An elegant review by Yuede et al. (2018) reported findings from studies of different exercise protocols in AD mouse models and discussed the interplay between stress and exercise in AD. Although research has focused on the effects of aerobic exercise (forced or voluntary exercise) in AD models (Nichol et al., 2007, 2008; Xiong et al., 2015), less is known about the impact of resistance exercise on this pathology. Our group and others have recently shown that resistance exercise may minimize the alteration in exploratory activity (Hashiguchi et al., 2020) and in cognitive function (Liu et al., 2020) in a transgenic mouse model for AD.

### Neurobiological Mechanisms

As with pre-adolescents and adolescents, changes in brain structure and function and improvement in several aspects of cognition have been observed in the adult population as result of better cardiorespiratory fitness. A meta-analysis conducted by Kramer and Colcombe (2018) has elegantly highlighted this issue. In younger adults, a 6-weeks exercise program was able to increase hippocampal volume (Thomas et al., 2016). In older subjects, 6 months of aerobic program (walking) induced increases in volume in frontal and temporal gray matter and white matter regions (Colcombe et al., 2006) and similar effects were reported by other studies (Erickson et al., 2011; Reiter et al., 2015). Although cardiorespiratory fitness induced by conventional physical training such as walking, jogging or cycling has been extensive reported to promote brain plasticity, a study conducted by Rehfeld et al. (2018) showed that dance training produced larger brain volumes compared to repetitive exercise in the elderly. Indeed, a recent systematic review highlights the impact of dance practice to induce brain plasticity (Teixeira-Machado et al., 2018).

Concerning white matter integrity, increases were reported in temporal and parietal regions in older subjects submitted to 1 year of an aerobic exercise program (Voss et al., 2013). Of note, better cardiorespiratory fitness in young and older adults has also been related with increases in hippocampal cerebral blood volume and memory improvement (Chapman et al., 2013), supporting the idea that regular exercise may build a vascular reserve against AD. With respect to neurotrophic factors, a link between BDNF, exercise, and cognition has been proposed during aging, suggesting that this association may have a critical role for the prevention and minimization of cognitive impairment during aging (Wang and Holsinger, 2018). Indeed, some authors have elegantly emphasized physical exercise as a means of restoring BDNF in this neurological condition, a finding that is also supported by animal studies (Wang and Holsinger, 2018). Essentially, aerobic fitness and serum BDNF levels in older adults have been correlated with increased hippocampal volume and better memory (Chaddock et al., 2010; Erickson et al., 2011). While the existing literature indicates the positive association of exercise and BDNF on cognition, further investigations are necessary in elderly and neurological subjects.

In pre-clinical studies, positive associations between exercise and cognition in adulthood and several possible neurobiological mechanisms have been proposed for the improvement of cognitive reserve. Extensive literature has reported that exercise can increase adult hippocampal neurogenesis (for a review see van Praag, 2008) and hypothalamus-pituitary-adrenal axis regulation (Wang et al., 2014), suppress apoptosis (Kim et al., 2010), reduce oxidative stress and mitochondrial dysfunction, modulate cytokine signaling (Speisman et al., 2013), increase vascular density (Ding et al., 2006) and production of BDNF and IGF-1 (Vaynman and Gomez-Pinilla, 2005), alter brain morphology (Stranahan et al., 2007) and electrophysiological properties of neurons (van Praag et al., 1999). These findings on brain plasticity induced by exercise are believed to support the improvement in learning and cognitive function.

The above-mentioned mechanisms revealed to be induced by exercise in the healthy population to improve cognitive performance could also be applied in aging and neurological disorders. In AD, some proposed mechanisms in mice models include increased BDNF expression, reduced inflammation, reduced amyloid deposition and phosphorylated tau accumulation (for a review see McGurran et al., 2019). For instance, reduced oxidative stress and Aβ scores were related to better working memory using three types of exercise for 6 weeks (aerobic, resistance, and combined exercises—aerobic + resistance) (Özbeyli et al., 2017).

### Exercise in Other Neurological Disorders

Besides the neuroprotective effects against aging, and an extensive number of studies looking at the prevention or minimizing of cognitive decline and dementia, regular physical exercise has had a positive influence in other neurological diseases. Here, we will focus on some common neurological disorders such Parkinson's, Epilepsy, and Stroke.

### Parkinson's Disease (PD)

PD is the second most common neurodegenerative disease worldwide, affecting 1% of the elderly population. A considerable number of scientific studies have highlighted the role of exercise in reducing the risk of PD, highlighting its contribution to resilience in PD. Moderate to vigorous physical sport/activities including tennis, biking, swimming, heavy housework in mid- to later life have been associated with a lower risk of PD (Xu et al., 2010). Of note, in a prospective study, the intensity of physical activity was an influential factor for better resilience in PD. Vigorous, but not moderate, physical activity was related to a 50% lower risk of PD in men (Chen et al., 2005). Thus, an elegant cross-sectional large-scale study with 9,676 elderly participants reported physical exercise as the best protective factor against PD (Zou et al., 2015). More recently, a large meta-analysis of more than half a million adults reported an association between high levels of moderate to vigorous activity and low risk of developing PD (Fang et al., 2018). Interestingly, while Yang et al. (2015) found that a medium level of daily total physical activity lowered the risk of Parkinson's disease, Logroscino et al. (2006) did not support the finding that physical activity protects against PD. As pointed out by Yang et al. (2015), to evaluate physical activity/exercise level, epidemiological studies have utilized different activities to measure moderate to vigorous exercise, such as leisure time, household, or energy expended on physical activity in kilocalories per week, which can produce different outcomes. Therefore, one must be cautious when analyzing these outcomes.

Although there is beneficial evidence of a decrease in the risk of PD, the influence of physical exercise in slowing the progression of the disease has not been completely explored. From few clinical trials, positive effects have been observed in PD resilience. Robottom et al. (2012) demonstrated that resilience in PD was associated with better health-related quality of life and reduced disability and non-motor symptoms (less apathy, depression, fatigue). Improvement in motor symptoms and physical functioning in people with PD was reported in a review which focused on the long-term effects of exercise (duration lasting at least 12 weeks) (Mak et al., 2017). Besides the classical motor symptoms, behavioral and cognitive symptoms are common in PD and positive results from exercise programs in several cognitive domains have been found in the literature (da Silva et al., 2018). Authors have reported improvement in the executive functions after aerobic (Altmann et al., 2016) or resistance training (Silva-Batista et al., 2016). With regard to the concept that resilience is an active process, the reduced disability and non-motor symptoms observed in PD could be minimized through regular physical exercise.

Regarding pre-clinical data, a number of studies have indicated that different types of aerobic exercise can improve cognitive function in PD. This positive effect has been investigated in exercise training applied before, during, and after parkinsonism-inducing treatment. Exercise can enhance the memory of rats by reducing cognitive impairment in a treadmill or swimming exercise protocol (Goes et al., 2014; Viana et al., 2017). In a voluntary exercise protocol, short-term exercise was effective in reducing cognitive deficits and depressive behavior and long-term exercise minimized the progression of motor symptoms (Hsueh et al., 2018; Mul et al., 2018).

### Neurobiological Mechanisms

In PD, limited information on neurobiological mechanisms has been reported in humans. Growing evidence suggests that physical exercise can minimize dopaminergic neuronal damage within basal ganglia motor circuits. In an *in vivo* study, the availability of the dopamine transporter by using [11C]CFT was reduced in the putamen after 1 h of strenuous walking in normal subjects but not in PD patients, indicating that this abnormal activation might be linked to the pathophysiology of the parkinsonian gait (Ouchi et al., 2001). In this regard, BDNF exerts an important influence in the mesolimbic dopaminergic pathway and altered BDNF levels have been observed in PD pathology. In post-mortem analysis, reduced expression of BDNF protein was observed in the *substantia nigra pars compacta* of PD patients compared to control subjects (Parain et al., 1999). Thus, blood analysis showed decreased serum BNDF levels in PD patients when compared with controls (Scalzo et al., 2010). In line with this, a systematic review and meta-analysis reported reduced serum BDNF levels in PD patients compared to healthy controls in spite of the presence of co-morbidities such depression among non-depressed and depressed PD patients (Rahmani et al., 2019). The physiological mechanisms of BDNF changes induced by exercise have been proposed in a systematic review and meta-analysis which reported increased BDNF blood levels following a physical exercise program in PD patients (Hirsch et al., 2018). In sum, the mechanisms and possible directions for neuroprotection in PD have been significantly explored, but more research is needed on the influence of exercise in this scenario in humans.

Pre-clinical investigations have revealed important effects of exercise on the nigrostriatal pathway, resulting in increasing extracellular dopamine release and reduction of striatal dopamine loss terminals (Petzinger et al., 2013). In part, these benefits are supported to facilitate DA neurotransmission and some studies support these affirmations. For instance, 4 weeks of swimming prevented the decrease of dopamine and its metabolites DOPAC and HVA levels induced by 6-OHDA in the striatum of mice and restored long-term memory in the object recognition test (Goes et al., 2014). Moreover, 2 weeks of treadmill reduced nigrostriatal dopaminergic cell loss, increased cell proliferation in the hippocampal dentate gyrus, and minimized short-term memory impairment in 6-OHDA-induced Parkinson's rats (Cho et al., 2013).

### Epilepsy

Epilepsy is another common neurological condition affecting over 70 million people worldwide. It is characterized by an enduring predisposition to generate spontaneous epileptic seizures, presenting several neurobiological, cognitive, psychological, and social consequences (Fisher et al., 2014). A number of factors such seizure control and reduced depression and anxiety can impact positively on the quality of life of people with epilepsy. These protective factors could be characterized as resilience in the context of epilepsy. Thus, regular exercise over the life course has been associated with resilience to developing epilepsy. In this scenario, some human investigations have explored whether previous physical exercise practice is able to reduce the incidence of epilepsy. For instance, a large and population-based cohort, consisting of 1,173,079 men, over a long observation period (up to 40 years) by a Swedish group showed that low cardiovascular fitness evaluated at age 18 was associated with a risk of presenting epilepsy later in life (Nyberg et al., 2013). Reinforcing the above finding, a more recent study conducted by Ahl et al. (2019) compared the incidence of epilepsy over 20 years in a large number of participants of a long-distance Swedish cross-country ski race (Vasaloppet) with the incidence of their match controls in a register of the Swedish population. They found up to 40–50% lower incidence of epilepsy in cross-country ski racers before retirement. Notably, data sub-analyses demonstrated that faster ski racers had a significant lower incidence of epilepsy compared to those with a slower finishing time. Both above findings are indicative that regular physical exercise and better physical fitness can be protective against developing epilepsy.

Physical exercise also plays a positive role after epilepsy is established. Factors such as social functioning, ability to work, stigma, prejudice, and adjustment to seizures significantly reduce the quality of life of people with epilepsy and are generally associated with depression and anxiety disorders. From the limited number of prospective studies in humans, a reduced number of seizures, reduced anxiety and depression symptoms, improvement of quality of life together with better capacity to cope with stress are observed after exercise programs (Bjorholt et al., 1990; Roth et al., 1994; McAuley et al., 2001; Vancini et al., 2013). Certainly, physical exercise has been suggested as a potential candidate for stress reduction in people with epilepsy (Arida et al., 2009). The influence of exercise on the reduction in seizure frequency or seizure susceptibility is an important factor in this picture. Seizure frequency has been negatively associated with cognitive function (Tromp et al., 2003; Hoppe et al., 2007). It has been demonstrated that people with temporal lobe epilepsy, the most common form of epilepsy, with low seizure frequency presented better performance on tests of anterograde memory compared to those with high seizure frequency (Voltzenlogel et al., 2014). In line with this, clinical studies have reported that physical exercise programs improve seizure control (Eriksen et al., 1994; Nakken, 1999). However, the influence of physical exercise on cognition is poorly investigated. To our knowledge, only two studies have explored the influence of an exercise program on cognition in people with epilepsy. Children with benign epilepsy submitted to ten supervised exercise sessions for 5 weeks showed significant improvements in attention tests (Eom et al., 2014). Although this is an encouraging result, the study presented a small sample, no control group, and only a short period of exercise intervention. Thus, most of them were seizure-free and receiving monotherapy. In a recent randomized study, adults with epilepsy submitted to 12 weeks of a combined exercise program presented an improvement in some domains of cognitive function such attention and language tasks (Feter et al., 2020). Although further research in humans is needed, the efficacy of exercise programs in improving cognitive functions in animal models of epilepsy have been very positive and play a beneficial role in increasing resilience to developing epilepsy.

Findings concerning exercise and cognition have been positive in different models of epilepsy. For instance, a 30-days swimming exercise reduced learning and memory deficits in rats submitted to a kainite acid model (Gorantla et al., 2016). Two types of exercise, voluntary wheel running and swimming, reduced seizure frequency and improved cognitive performance, measured by the object recognition test and the passive avoidance test (Lin et al., 2019). Recently, we demonstrated that resistance exercise was able to decrease the number of seizures, which was associated with a reduction in memory impairment (de Almeida et al., 2017). Promising results have been found with regard to investigations about the effects of exercise on cognitive changes in animals submitted to *status epilepticus* earlier in life. Rats submitted to penicillin-induced recurrent *epilepticus* in early life and then exercised during the final period of adolescence (P49-P54) presented better performance in the water maze test than their controls (Ni et al., 2009). In addition, cognitive impairments induced by lithium-pilocarpine-induced *status epilepticus* at weaning was decreased in animals housed in environmental enrichment with wheel running (Fares et al., 2013). Thus, an exercise program in rats conducted during the period of post-natal brain development reduced seizure susceptibility later in life, indicating that exercise undertaken in early life may build a neural reserve against brain disorders (Gomes da Silva et al., 2011). The authors suggest that physical exercise during early periods of life may provide an adequate development of neural circuitry, which can support greater brain damage in later life. Collectively, these studies reinforce the neuroprotective and rehabilitative influence of exercise in epilepsy.

### Neurobiological Mechanisms

With epilepsy, we can only make suppositions regarding the effects of exercise in humans. Some possible mechanisms are modulation of the neurotransmitter system and changes in metabolic, neuroendocrine, and growth factors. Cellular and molecular events underlying epilepsy can impair growth factor signaling in the brain and current evidence has associated BDNF with the pathophysiology of epilepsy in humans. Increased levels of BDNF have been found in the surgically resected temporal neocortex (Takahashi et al., 1999) or hippocampus of patients with TLE, demonstrating that epileptic activity may increase BDNF levels and its gene expression (Murray et al., 2000). Conversely, in the periphery, decreased levels of plasma (LaFrance et al., 2010) and serum BDNF have been reported in adult patients with epilepsy (Hong et al., 2014). Considering that regular exercise has been known to reduce neuroinflammation, oxidative stress, and excitotoxicity (Moylan et al., 2013), it can be suggested that exercise might modulate these effects in this condition, therefore building brain resilience against epilepsy.

Although there is no reported effect in epilepsy patients, positive outcomes have been found in animal models of epilepsy. Modulation of excitatory/inhibitory systems and neurotrophic factors has been demonstrated in several epilepsy models. Reduced levels of synaptic-related proteins and GABAergic function in epileptic animals were reversed by exercise, which was associated with improved cognitive function and reduced seizure frequency (Lin et al., 2019). Recently, we examined the influence of exercise on the activation of downstream proteins related to BDNF-TrkB receptor. Aerobic exercise increased hippocampal BDNF expression, restored the overexpression of full-length TrkB and truncated-TrkB isoforms to control levels, and altered the hippocampal activation of some proteins linked to BDNF-TrkB intracellular signaling (de Almeida et al., 2018) and resistance exercise restored to control levels the altered BDNF levels and ERK and mTOR activation in rats with epilepsy (de Almeida et al., 2017). Thus, improved learning and memory in rats with epilepsy housed in an enriched environment with wheel running was accompanied by increased hippocampal neurogenesis and BDNF and VEGF transcripts (Fares et al., 2013).

### Stroke

Stroke is the second leading cause of mortality and the principal cause of physical disability around the world. Despite extensive efforts, the development of preventive or rehabilitative treatment has not been totally effective (Moskowitz et al., 2010). Among therapies for this condition, physical activity/exercise has been proposed in a large number of investigations. The literature shows that regular practice of physical exercise is inversely related to stroke risk. Not only in stroke prevention, but also in stroke recovery in physical and cognitive tasks (Han et al., 2017).

A large amount of research has reported an inverse association between cardiorespiratory fitness and stroke. In one investigation of 16,000 men, those with moderate or high fitness presented lower risk of stroke mortality compared to low fitness individuals (Lee and Blair, 2002). In another large cohort study with women during a mean follow-up of 11.9 years, walking was related with lower risks of total, ischemic, and hemorrhagic stroke (Sattelmair et al., 2010). In accordance with the above findings, a meta-analysis by Lee et al. (2003) found that active individuals presented lower risk of stroke incidence and between the two categories of active subjects, moderately active and highly active, individuals had a 20 and 27% lower stroke risk, respectively, than the low active subjects. Although vigorous exercise is more effective, it seems that people who usually exercise at moderate levels can also see some benefit in preventing stroke. Considering that participating in moderate activity applies to the much of the population, this issue may be better explored in future studies.

The benefits of exercise have also been supported by various studies in individuals who have had a stroke. For instance, there is robust evidence for the beneficial effects of exercise on improving cardiorespiratory fitness and mobility after stroke. This evidence is based on a Cochrane Systematic Review which included 2,797 participants from 58 studies to examine the influence of physical exercise on stroke recovery (Saunders et al., 2016). The supportive effect of exercise on rehabilitation post-stroke has also been documented in clinical guidelines (Billinger et al., 2014). In addition to the above benefits, improvement in cognitive function has been reported in several controlled trials applying structured physical training to stroke patients (Blanchet et al., 2016; Ihle-Hansen et al., 2019). A meta-analysis of randomized controlled trials selecting studies with a duration of training >4 weeks showed a positive impact of exercise on some measurements of cognitive performance such as attention/processing speed following a stroke (Oberlin et al., 2017). Taking into account that studies address different or combined interventions, i.e., different inclusion criteria across the studies such as physical activity (leisure time or recreational activity), exercise program or cardiorespiratory fitness, a study conducted by Brouwer et al. (2019) evaluated the influence of solely aerobic training on vascular and metabolic risk factors for stroke. A beneficial effect on systolic blood pressure and fasting glucose was observed after stroke compared to non-aerobic exercise. More investigations using physical training programs will provide a better perception of this intervention (aerobic exercise) for improving resilience to stroke. With this in mind, higher levels of physical activity and exercise are important factors in reducing the risk for cardiovascular diseases and consequently increasing resilience for stroke.

In addition to physical disability and cognitive impairments, social factors are also common in stroke patients, notably changes in depressive symptoms, and social desirability. In a survey with stroke survivors, feelings of depression, fatigue, and low motivation were found to be factors that reduced the desire to undertake physical activity, while self-determination and goal achievement were related with greater involvement in physical activities (Morris et al., 2012). Therefore, healthy habits early or during the life course might influence healthier behaviors and cognitive recovery after stroke.

In general, animal models of stroke have confirmed that aerobic exercise of moderate intensity seems to be beneficial to cognition. Indeed, increasing reports have indicated that early exercise is protective against stroke (Zhang et al., 2015) and can minimize memory impairment induced by ischemic brain injury (Sim et al., 2004, 2005). For instance, 2 weeks of aerobic exercise introduced 24 h after middle cerebral artery occlusion significantly induced spatial memory recovery (Yang et al., 2017). Although these are positive findings, it is important to note that the intervention time window and exercise intensity are critical to the outcome. In this regard, some studies have reported that low-intensity exercise induces better effects on a spatial learning task in a water maze test and on both object recognition and location tasks than high-intensity exercise (Shih et al., 2013; Shimada et al., 2013). Overall, animal data suggest that physical exercise plays a role in cognitive function, thereby enhancing cognitive reserve. With regard to pre-stroke, although there is little information on cognitive effects, animal studies have clearly demonstrated that pre-ischemic exercise may minimize stroke severity in functional motor outcomes (Ding et al., 2004).

### Neurobiological Mechanisms

Several mechanisms by which physical exercise exerts an effect in the prevention of and recovery from stroke are similar from those observed in the neurological diseases mentioned above. Acute ischemic stroke patients reporting a high level of physical activity prior to stroke were associated with greater VEGF expression and good outcomes after stroke (López-Cancio et al., 2017).

Many pre-clinical studies have examined the possible role of physical exercise in pre-conditioning and tolerance to cerebral ischemia. Exercise pre-conditioning-induced neuroprotection is mediated by multiple processes, such as maintenance of the integrity of the blood-brain barrier, anti-inflammatory effects, neovascularization, neurogenesis, and reduced excitotoxicity (Islam et al., 2017). For instance, data from animal models with cerebral ischemia or stroke submitted to exercise training have demonstrated positive neuroplasticity resulting in better cognitive performance, which can be attributed to increased expression of BDNF, synapsin-I, and post-synaptic density protein 95 (PSD-95) (Shih et al., 2013). Consistent with findings reported in health and in different brain diseases, it is supposed that BDNF plays an import role in this process because the improvement in spatial learning induced by exercise is reduced after the TrkB-BDNF receptor is blocked (Griesbach et al., 2009). Furthermore, better cognition in exercised animals has been associated with increased neurogenesis after cerebral ishemia (Luo et al., 2007) and increased antioxidant enzyme activity in the hippocampus in rats with bilateral common carotid artery occlusion (Cechetti et al., 2012). In sum, the pre-clinical findings clearly demonstrate that exercise has an important effect on metabolic and neurotrophic influences, which can explain its role in brain health and supportive action in terms of both resilience to and prevention of stroke.

## Conclusion

**Figure 2:**
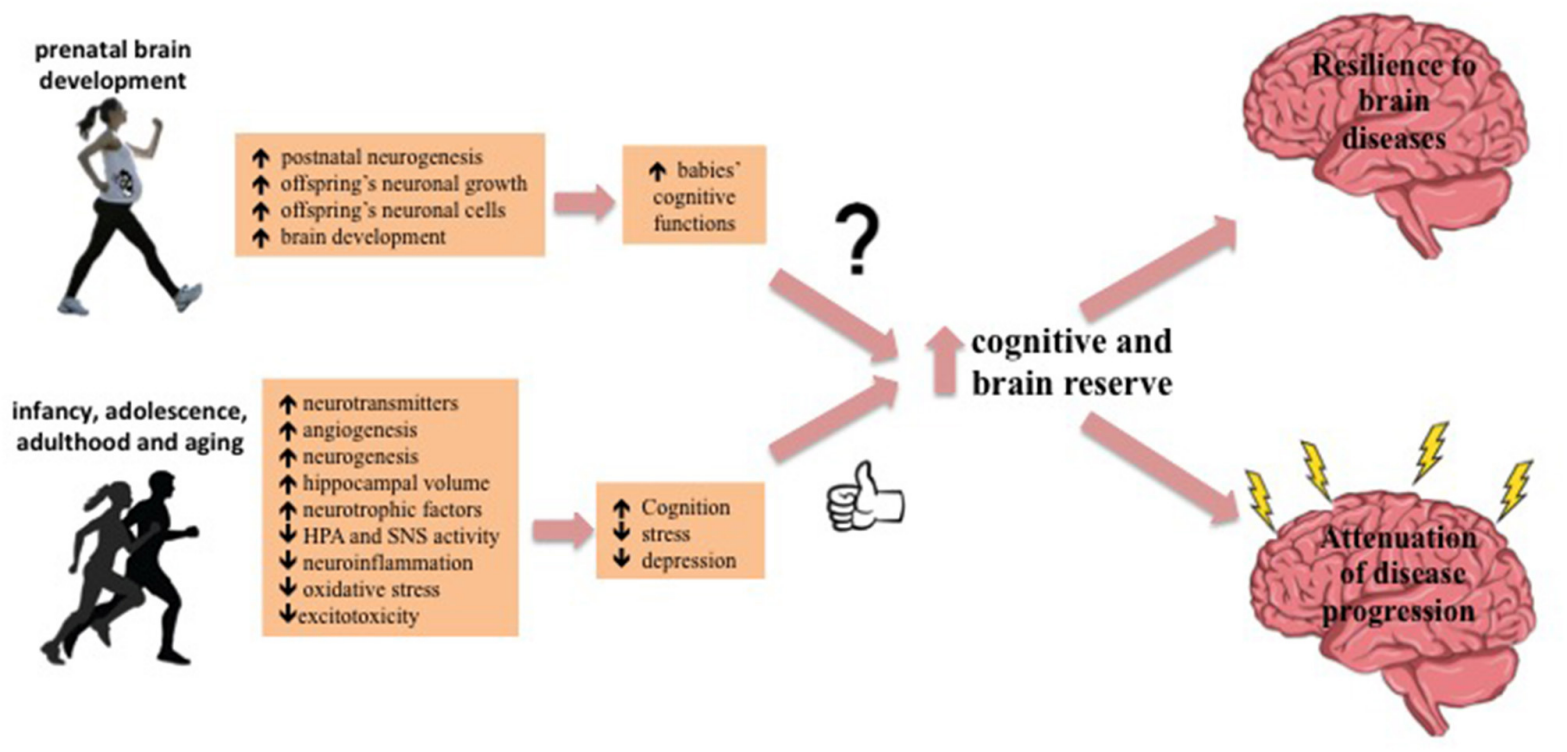
Model of how exercise can confers brain resilience. Exercise positively influences brain and cognitive reserve by multiple pathways, protecting against the consequences of stressful events, improving health, and reduce the risk of chronic disease.

Nowadays, we are constantly bombarded by media, physicians, and other health professionals to engage in physical/sports activities to reduce physical/psychological stress, improve our health, and reduce the risk of chronic disease. The literature has clearly demonstrated aerobic fitness as one of the best indicators of resilience. This is supported by evidence from a number of studies showing that physical fitness confers physiological and psychological benefits and protects against the development of stress-related disorders, as well as improves cognition and motor function that are a consequence of aging and of neurological disorders. Although we have learned about neurobiological mechanisms of physical fitness from the neuroplasticity and neuroprotection that confer resilience, these effects and mechanisms are diverse and complex and need to be further explored. However, we can summarize that exercise modulates several mechanisms that may increase brain health and counteract brain disorders (Figure 2). Exercise positively influences neuronal reserve by increasing BDNF expression which promotes neurogenesis and synaptic plasticity, reduces oxidative stress and inflammation, and enhances cerebral and peripheral blood flow, which stimulates angiogenic factors that lead to positive changes in the structure and morphology of brain vasculature. All these changes shape brain activity and serve as a buffer against stress-related disorders. Positive findings have also been observed concerning the effects of exercise on cognition and therefore cognitive reserve. However, several null findings, not reported here, should be analyzed with caution because different types of exercise intervention are confounders that can interfere with the studies' outcomes. The influence of exercise variables includes the duration, frequency, intensity, and length of exercise. To this point, several exercise regimens have been used to determine the optimal stimulus on brain resilience. However, the evidence for dose-response relationship between exercise and brain/cognitive reserve in different life stages is not well comprehensive. Research investigating different dose-parameters of exercise both in animal and human has generated inconsistent results. These conflicting results may be partially attributed to the diversity of classifications used for high or low exercise intensity, volume and type (aerobic or resistance or anaerobic) of exercise that can be beneficial for brain resilience (Wipfli et al., 2008; Vidoni et al., 2015; Kramer and Colcombe, 2018; Greene et al., 2019). For older population, a systematic review and meta-analysis reported that healthy subjects should perform both aerobic and anaerobic exercises, of moderate intensity, for at least three times per week and those with cognitive impairments, exercise with shorter duration and higher frequency to obtain better cognitive outcomes (Sanders et al., 2019). In general, the American College of Sports Medicine recommendations should be applied (ACSM American College of Sports Medicine, 2013).

While several models of physical activity or exercise may impact positively on brain resilience such yoga, dance, martial arts, etc., in this review we aimed to focus mainly on the effects of aerobic exercise of low and moderate intensity or resistance exercise. Thus, physiological markers including heart rate variability, blood pressure and cortisol might be regularly used as indicator of stress to determine the impact of exercise on brain resilience. Some examples of stress systems are the immune-inflammatory system, the HPA—axis and the autonomic nervous system (Meggs et al., 2016). Disturbance of these systems could lead to hyperactivity of the HPA-axis, sympathetic activation and systemic inflammation. However, there are still unanswered questions concerning (1) whether physical exercise in early life can prevent or delay cognitive decline in later life, (2) the effectiveness of exercise programs for individuals across the life span and for those with neurological diseases, and (3) how much exercise is necessary to gain beneficial effects on cognitive health. This is a field of research that deserves more attention. Thus, it is unquestionable that multiple interacting factors, not explored in this review, including nutritional status and the link between genes and environment, mediated by epigenetic changes that occur at critical periods of development may confer vulnerability or resilience to brain disorders. Overall, this review provides an insight into the current evidence of physical exercise's contribution to the increase in resilience and brain tolerance to aging, pathology, and insult.

## Author Contributions

RMA and LTM participated in the design of the work, the bibliographic review and writing of the manuscript. Both authors approved the submitted version.

## Conflict of Interest

The authors declare that the research was conducted in the absence of any commercial or financial relationships that could be construed as a potential conflict of interest.
